# Exploring the Interplay between Fatty Acids, Inflammation, and Type 2 Diabetes

**DOI:** 10.3390/immuno4010006

**Published:** 2024-03-01

**Authors:** Dequina A. Nicholas, Jacques C. Mbongue, Darysbel Garcia-Pérez, Dane Sorensen, Heather Ferguson Bennit, Marino De Leon, William H. R. Langridge

**Affiliations:** 1 School of Biological Sciences, University of California Irvine, Irvine, CA 92697, USA; 2 Department of Biological Sciences, School of Arts and Sciences, Oakwood University, Huntsville, AL 35896, USA; 3 Center for Health Disparities and Molecular Medicine, School of Medicine, Loma Linda University, Loma Linda, CA 11085, USA; 4 Division of Molecular Genetics and Microbiology, School of Medicine Alumni Hall, Loma Linda University, Rm 102, 11021 Campus Street, Loma Linda, CA 92350, USA; 5 Center for Perinatal Biology, Division of Physiology, Loma Linda School of Medicine, Rm A572, 11234 Anderson Street, Loma Linda, CA 92350, USA

**Keywords:** type 2 diabetes, inflammation, fatty acids, cytokines, adipose, immune cells

## Abstract

Around 285 million people worldwide currently have type 2 diabetes and it is projected that this number will be surpassed by 2030. Therefore, it is of the utmost importance to enhance our comprehension of the disease’s development. The regulation of diet, obesity, and inflammation in type 2 diabetes is believed to play a crucial role in enhancing insulin sensitivity and reducing the risk of onset diabetes. Obesity leads to an increase in visceral adipose tissue, which is a prominent site of inflammation in type 2 diabetes. Dyslipidemia, on the other hand, plays a significant role in attracting activated immune cells such as macrophages, dendritic cells, T cells, NK cells, and B cells to visceral adipose tissue. These immune cells are a primary source of pro-inflammatory cytokines that are believed to promote insulin resistance. This review delves into the influence of elevated dietary free saturated fatty acids and examines the cellular and molecular factors associated with insulin resistance in the initiation of inflammation induced by obesity. Furthermore, it explores novel concepts related to diet-induced inflammation and its relationship with type 2 diabetes.

## 1. Introduction

Diabetes mellitus can be categorized into three primary types: type 1, type 2, and gestational diabetes. Type 1 diabetes is a well-defined autoimmune condition characterized by the selective destruction of pancreatic islet β-cells, which are responsible for insulin secretion. Type 2 diabetes accounts for 90–95% of diabetes cases globally and represents a complex metabolic disorder primarily stemming from insulin resistance. Insulin resistance is a condition where the body can produce insulin, but cells cannot efficiently utilize it. Gestational diabetes affects 3–8% of pregnant women and may result from pregnancy-related hormones or insufficient insulin production. This review will be focused on type 2 diabetes and obesity-induced inflammatory immune mechanisms implicated in insulin resistance [1].

Worldwide, approximately 285 million people have been diagnosed with type 2 diabetes [1,2]. In the U.S., healthcare costs are reported to be over 245 billion dollars annually [3]. The rising prevalence of type 2 diabetes is linked to a range of risk factors. Genetic factors including an enhanced appetite and a tendency to accumulate abdominal fat can elevate the risk of developing type 2 diabetes. However, it is generally believed that modifiable factors like a high-energy or high-fat diet, as well as reduced physical activity are the primary contributors to the development of this disease [4]. These environmental factors seem to promote inflammation, which is a third significant risk factor for the development of type 2 diabetes. Persistent, low-level inflammation in tissues, particularly in visceral adipose tissue, is associated with obesity and the onset of type 2 diabetes [5,6]. The following sections of this review will detail the impact of high-fat diets on inflammation in visceral fat and its role in the development of type 2 diabetes.

## 2. Diet, Adipose Tissue, and Type 2 Diabetes

### 2.1. Adipose Tissue in the Obese State

**Figure 1.**
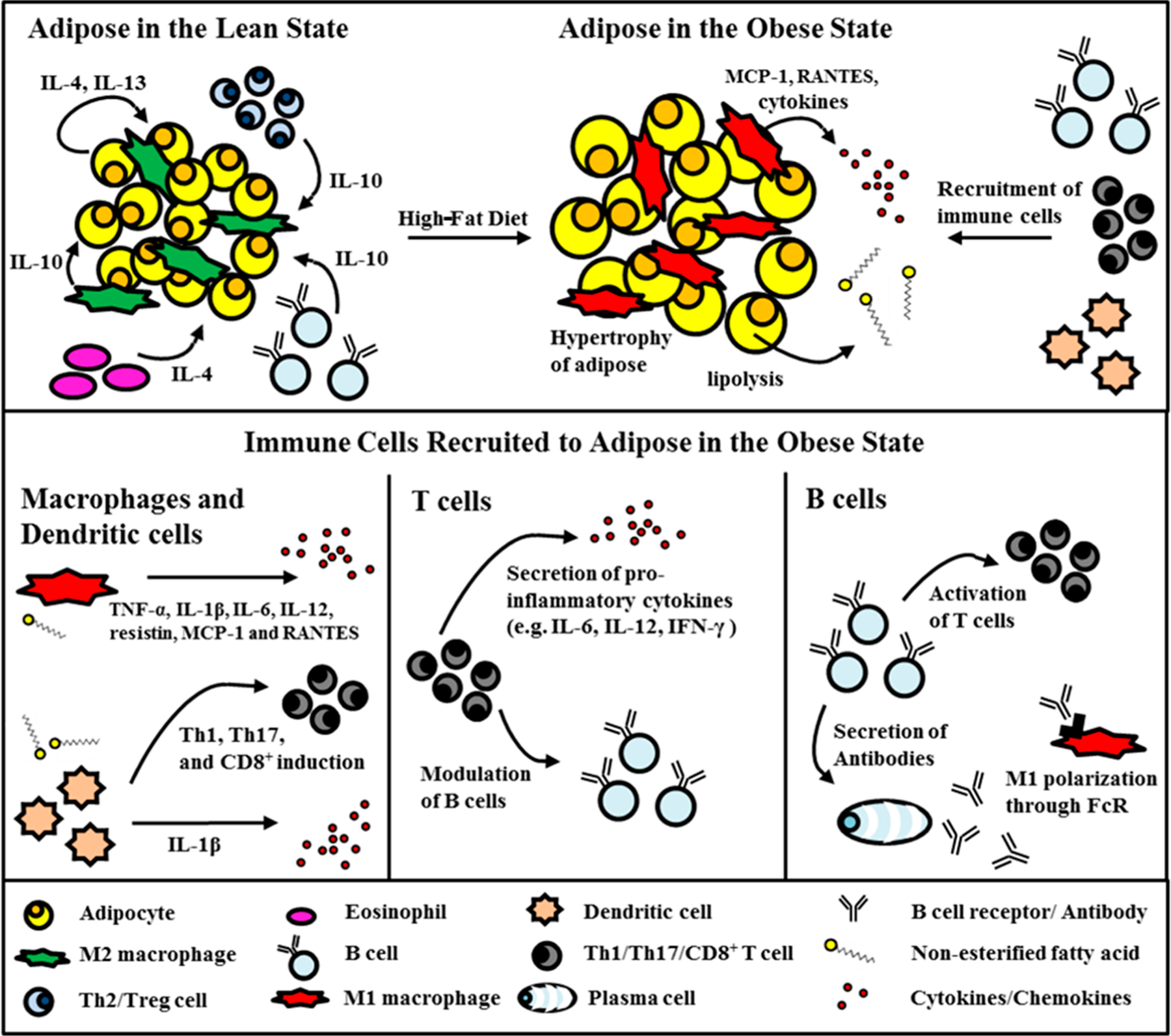
Overview of Inflammation Induced by Obesity: In a state of leanness, resident macrophages maintain the M2 phenotype, a process driven by a combination of factors including IL-10 from Treg and Breg cells, IL-4 from eosinophils and adipocytes, and IL-13 from adipocytes. These factors collectively polarize macrophages toward the M2 phenotype, which, in turn, secrete IL-10. However, in obesity, adipocytes undergo hypertrophy, form necrotic centers due to hypoxia, and experience increased lipolysis. These conditions lead to the polarization of macrophages toward the M1 phenotype, characterized by the secretion of pro-inflammatory cytokines and chemoattractant molecules that recruit immune cells to adipose tissue. It is likely that the heightened presence of saturated fatty acids resulting from lipolysis activates macrophages and dendritic cells through Toll-like receptor (TLR) signaling. Activated M1 macrophages then secrete cytokines that promote the maintenance of Th1, Th17, and CD8+ T cells. Dendritic cells release IL-1β, which contributes to insulin resistance and guides the differentiation of naive T cells into pro-inflammatory subsets. Furthermore, Th1, Th17, and CD8+ T cells themselves release classical pro-inflammatory cytokines, which further drives the polarization of macrophages toward the M1 phenotype. Additionally, T helper cells provide stimulation to B cells, leading to robust antibody responses. B cells can serve as antigen-presenting cells, activating pro-inflammatory T cell subsets. After receiving T cell help, B cells can differentiate into plasma cells that secrete antibodies, capable of polarizing macrophages to the M1 phenotype and activating macrophages through Fc receptor binding.

For numerous years, extensive research has been conducted to investigate the connection between an unhealthy diet, obesity, and the development of type 2 diabetes. Chronic, low-grade inflammation appears to be the common factor linking these three elements. Adipose tissue, now acknowledged as a significant endocrine organ rather than a passive tissue primarily for insulation and warmth, has been recognized as the primary site where inflammation is triggered by obesity. Adipocytes, primarily responsible for storing energy in the form of lipids, have a secondary role as an endocrine organ, releasing hormones like leptin, estrogen, resistin, and pro-inflammatory cytokines like TNF-α. More recently, it has been revealed that adipocytes also possess direct immune functions, as they express CD40 and MHCII, which play a role in activating T cells in the context of type 2 diabetes [7,8]. Additionally, adipocytes have the capability to release pro-inflammatory cytokines like TNF-α and IL-1β, which are factors contributing to the development of insulin resistance [9–11]. In periods of leanness, adipocytes can produce cytokines such as IL-4 and IL-13, which possess the capacity to promote the differentiation of macrophages into the anti-inflammatory M2 phenotype (Figure 1) [12]. Conversely, an increase in visceral adipose tissue leads to persistent inflammation. In individuals with obesity, there is a significant rise in the release of pro-inflammatory substances, including non-esterified fatty acids (FAs) and cytokines [13]. In an insulin-resistant state, adipocytes undergo high levels of lipolysis and release free fatty acids (Figure 1). Furthermore, when prompted by TNF-α, such as during inflammation induced by obesity, adipocytes generate and release the chemoattractant molecule CCL5. This molecule has the capability to attract and recruit T cells to fat tissues [14].

### 2.2. The Role of Fatty Acids in Triggering Insulin-Secreting Beta-Cells’ Failure

The adverse impacts of sustained elevation in free fatty acid (FFA) levels on glucose regulation are termed lipotoxicity, with the combined exposure to elevated glucose potentially exacerbating these effects, known as synergistic glucolipotoxicity.

Extended exposure of isolated islets or insulin-secreting cells to heightened levels of fatty acids (FAs) is linked with several detrimental effects, including the inhibition of glucose-stimulated insulin secretion (GSIS), the decreased expression of insulin genes, and the initiation of apoptosis. Rat islets cultured for seven days under conditions of elevated FFAs display characteristic apoptotic events such as DNA fragmentation, heightened caspase activity, formation of ceramide, and upregulation of apoptotic genes, contrasting starkly with untreated rat islets [15]. Exposure to long-chain saturated FFAs, such as palmitic acid (16:0), stearate (C18:0), arachidate (C20:0), and linocerate (C24:0), triggers ceramide accumulation through the actions of serine palmitoyl transferase and ceramide synthase (CerS). However, this effect is not observed with shorter saturated FFAs, such as myristate (C12:0), or unsaturated FFAs [16,17].

De novo ceramide synthesis is proposed as a mediator of FFA-induced toxicity in beta-cells. The elevated expression of CerS4 exacerbates palmitic acid (PA)-induced ceramide accumulation, leading to enhanced apoptosis by generating additional toxic ceramide species, including C18:0, C22:0, and C24:1 [18]. It has always been known that blocking de novo ceramide synthesis through the use of inhibitors targeting serine palmitoyl transferase (e.g., L-cycloserine) or ceramide synthase (e.g., fumonisin-B1) mitigates beta-cell apoptosis induced by FFAs and reduces hyperglycemia [15,19].

FFAR1 (GPR40) is activated by medium- and long-chain FFAs (especially ecosatrienoic acid (C20:3)) and facilitates glucose-stimulated insulin secretion (GSIS) in pancreatic beta-cells [20].

The insulin secretory effect of FFAs on beta-cells diminishes with the loss of FFAR1 function. Steneberg et al. illustrated that FFAR1 deficiency shields mice from obesity-induced hyperinsulinemia, hyperglycemia, and glucose intolerance. Conversely, overexpression of FFAR1 in the beta-cells of mice results in an impaired beta-cell function and the onset of diabetes [21].

Additionally, studies have shown that increased levels of saturated FFAs negatively affect insulin gene transcription. In isolated islets, research revealed that simultaneous exposure to elevated levels of glucose and palmitate inhibits the expression of the transcription factor MafA and the nuclear translocation of the transcription factor PDX-1 [22–25].

The endoplasmic reticulum (ER) plays a pivotal role in lipid biosynthesis, calcium storage, and the synthesis and proper folding of secreted proteins. When unfolded or misfolded proteins accumulate in the ER lumen, this condition is termed ER stress, triggering the unfolded protein response, also referred to as the ER stress response. Palmitate induces ER stress by disrupting ER folding capacity, leading to an overload of misfolded proteins within the ER of pancreatic beta-cells [26].

An imbalance between the formation of reactive oxygen species (ROS) and the cellular antioxidant defenses can result in oxidative stress. Free fatty acids (FFAs) and excessive glucose are powerful inducers of ROS through distinct mechanisms. Long-chain FFAs (with more than 14 carbons) are inefficient substrates for mitochondrial β-oxidation. Instead, they undergo peroxisomal β-oxidation and are then transported to mitochondria for further breakdown. The peroxisomal β-oxidation of palmitate produces hydrogen peroxide (H_2_O_2_) [27].

### 2.3. Dietary Fatty Acids and Type 2 Diabetes

As mentioned earlier, one significant risk factor for the development of type 2 diabetes is a high-fat diet, especially one rich in saturated fats. Such diets contribute to elevated levels of long-chain saturated fatty acids. Increased dietary intake of these saturated fatty acids, such as palmitic and stearic acids, has been linked to the initiation of inflammation and the development of type 2 diabetes [28–30]. Palmitic acid (PA) is a particularly abundant fatty acid and is predominantly found in foods like meats, dairy products, and palm oils. Given the prevalence of these fatty acids in the Western diet, the composition of dietary fatty acids plays a substantial role in determining the relative presence and storage of fatty acids within the body’s tissues [31]. The ready accessibility of processed foods, combined with excessive calorie consumption, can potentially lead to obesity. This, in turn, can result in elevated levels of fatty acids in both the bloodstream and body tissues. These elevated fatty acid levels may be a contributing factor to the development of insulin resistance [31]. Elevated levels of non-esterified fatty acids (NEFAs) in the bloodstream are associated with metabolic dysfunction, obesity, and an elevated risk of developing type 2 diabetes [32–35]. Numerous studies have consistently demonstrated that NEFAs can induce insulin resistance in both the liver and skeletal muscle [36–38]. Too much serum non-esterified FAs are also known to induce the inflammation responsible for generating systemic insulin resistance [39,40].

Various potential pathways have been suggested for the advancement of clinical treatments utilizing FFA receptor agonism. Among these is the exploration of co-therapeutic strategies combining FFA receptor agonists with existing type 2 diabetes mellitus (T2DM) therapies. For instance, AS2575959, functioning as an FFA1 agonist, demonstrates synergistic effects when used alongside a DPP-IV inhibitor, leading to enhanced glucose homeostasis [41]. DS-1558, functioning as an FFA1 agonist, exhibits synergistic effects when combined with exendin-4, leading to improved glucose homeostasis in diabetic mice [42].

### 2.4. Saturated Fatty Acid Stimulation of Immune Cells

While elevated levels of free fatty acids can have a detrimental effect on metabolic balance, it is important to note that not all non-esterified fatty acids are the same. Specifically, saturated fatty acids obtained from dietary triglycerides or released through adipose tissue lipolysis are typically regarded as pro-inflammatory. The primary mechanism that has gained prominence in explaining how saturated fatty acids might influence the immune system revolves around the activation of a pathogen recognition receptor known as Toll-like receptor 4 (TLR4) [43]. Numerous studies have provided evidence to support the idea that Toll-like receptor 4 (TLR4) serves as a connection point between innate immunity and the development of insulin resistance induced by fatty acids [43]. Research has demonstrated that saturated fatty acids can activate macrophages by interacting with Toll-like receptor 2 (TLR2) and Toll-like receptor 4 (TLR4) [44–46]. This finding holds significance because Toll-like receptor 4 (TLR4) signaling plays a crucial role in the maturation of M1 macrophages, which are responsible for the secretion of pro-inflammatory molecules such as TNF-α, IL-1, IL-6, IL-12, and inducible nitric oxide synthase (iNOS), as well as chemoattractants like monocyte chemoattractant protein 1 (MCP-1) and RANTES, in the inflamed adipose tissue during the development of type 2 diabetes [6]. It has also been demonstrated that saturated fatty acids can activate both adipocytes and microglia through Toll-like receptor (TLR) signaling [47–49]. Moreover, our research laboratory has provided evidence that palmitic acid (PA) activates dendritic cells, prompting the secretion of IL-1β by triggering Toll-like receptor 4 (TLR4) activation. This observation aligns with the notion that mice with TLR4 mutations or deficiencies are shielded from the inflammatory consequences of a high-fat diet abundant in palmitic acid [50,51]. Lastly, it is important to note that Toll-like receptor 4 (TLR4) in hematopoietic cells plays a critical role in the development of insulin resistance in adipose tissue [43,52]. While it has been firmly established that fatty acids can disrupt Toll-like receptor 4 (TLR4) signaling, the precise mechanism by which saturated fatty acids interact with components of the TLR4 pathway remains to be fully elucidated.

## 3. Visceral Fat Inflammation in Type 2 Diabetes

Visceral adipose tissue (VAT) has, indeed, emerged as a pivotal site for inflammation linked to the development of type 2 diabetes [6]. There are various perspectives on the initiation of adipose tissue inflammation. Nevertheless, the prevailing belief is that a high-fat diet, followed by an increase in visceral adipose tissue, is the primary trigger for the recruitment of immune cells. Obesity leads to the formation of apoptotic centers within hypertrophic adipocytes [9,53], leptin secretion, unfolded protein responses activated by ER stress [54], secretion of MCP-1 from hypoxia-activated macrophages, and lipolysis that releases free FAs, which ultimately lead to immune cell infiltration, inflammation, and insulin resistance [55]. The infiltration of immune cells into visceral adipose tissue gives rise to a state of low-grade, chronic inflammation. This leads to the secretion of pro-inflammatory cytokines, which have the potential to induce insulin resistance, affecting both local and systemic insulin sensitivity (Figure 1). While the exact sequence of immune cell infiltration is a subject of debate, it is increasingly clear that a diverse array of immune cells plays a role in the inflammation of visceral fat [6]. In the subsequent section, we will delve into the primary subsets of immune cells that have been implicated in type 2 diabetes and also explore the emerging roles of less well studied immune cell types.

### 3.1. Monocytes/Macrophages

Macrophages have garnered the most attention among immune cells in the context of visceral adipose tissue (VAT) inflammation. These macrophages, which serve as antigen-presenting cells (APCs), are recognized as the primary source of cytokine secretion during chronic inflammation. In the lean state, VAT is predominantly populated by M2 macrophages, also known as alternatively activated or anti-inflammatory macrophages [6]. It is widely acknowledged that M2 macrophages play a crucial role in tissue surveillance, contribute to tissue remodeling, and help preserve insulin sensitivity in visceral adipose tissue (VAT). The polarization of macrophages into the M2 phenotype is induced by the presence of interleukins such as IL-4, IL-10, and IL-13 [56]. M2 macrophages release anti-inflammatory cytokines such as the IL-10 and IL-1 receptor antagonists, which help protect against insulin resistance [56]. Resident macrophages have the ability to identify deceased adipocytes and can become activated by fatty acids (FAs) through Toll-like receptor 2 (TLR2) and Toll-like receptor 4 (TLR4) [57]. TLR4 is responsible for inducing the polarization of macrophages into the M1 phenotype, which is associated with classical activation and pro-inflammatory characteristics. These M1 macrophages originate from monocytes that are recruited to visceral adipose tissue (VAT) during inflammation induced by obesity [58]. The influx of M1 macrophages disrupts the equilibrium between M1 and M2 macrophages, leading to inflammation. The secretion of pro-inflammatory molecules like TNF-α, IL-1β, IL-6, IL-12, resistin, MCP-1, and RANTES by M1 macrophages attracts additional monocytes and impedes insulin signaling, often by promoting serine phosphorylation [5,59]. The infiltration of macrophages into visceral adipose tissue (VAT) plays a pivotal role in inflammation induced by obesity. In fact, blocking the infiltration of CD11b myeloid cells, particularly macrophages, has been shown to prevent the detrimental effects of a high-fat diet in mice [60]. In additional evidence, improving glucose tolerance in diet-induced obese (DIO) mice was achieved by eliminating M1 macrophages through the targeted use of a diphtheria toxin on CD11c+ cells that are sensitive to diphtheria toxins [61]. In summary, the fundamental role of macrophages in the inflammation of visceral adipose tissue (VAT) is well-established and well-defined [58]. Nevertheless, the initial trigger that prompts the recruitment of monocytes to visceral adipose tissue (VAT) remains a subject of ongoing investigation and research.

### 3.2. Dendritic Cells

Dendritic cells (DCs) are considered professional antigen-presenting cells and serve a critical role in bridging the gap between the innate and adaptive immune responses. They achieve this by presenting antigens on major histocompatibility complex class II (MHCII) molecules to T cells [62]. The role of dendritic cells (DCs) in inflammation induced by obesity is not well understood, primarily because it is commonly believed that this type of inflammation is primarily driven by innate immune responses rather than adaptive immune responses [63]. Nonetheless, research has indicated that both the adaptive immune response and dendritic cells (DCs) play a role in inflammation induced by obesity [6]. An investigation focused on depleting CD11c+ adipose tissue macrophages in obese diabetic mice has suggested that dendritic cells (DCs) could potentially have a significant role in the development of insulin resistance induced by obesity [61]. In this study, the researchers observed that the depletion of CD11c+ cells in mice led to an improvement in obesity-induced insulin resistance and a reduction in pro-inflammatory cytokines within the adipose tissue. While these outcomes were initially attributed to adipose tissue macrophages, it is important to note that the ablation of CD11c+ cells, a marker commonly associated with classical dendritic cells, also resulted in the depletion of circulating dendritic cells (DCs), which actually outnumber adipose tissue macrophages [64]. Hence, this study also suggests that circulating CD11c+ dendritic cells (DCs) may have a role in obesity-induced inflammation. In line with this interpretation, two separate research groups have demonstrated an increase in the numbers of dendritic cells within visceral adipose tissue (VAT) in mice, during obesity [65,66]. Additionally, it is worth noting that CD103 dendritic cells (DCs), a specific subset recognized for its significance in the differentiation of regulatory T cells (Treg cells), are found to be reduced in the visceral adipose tissue (VAT) of obese mice [67]. Interestingly, Bertola et al. demonstrated that dendritic cells (DCs) isolated from the visceral adipose tissue (VAT) of obese mice had the capacity to induce the differentiation of naïve T cells into Th17 cells in vitro. Th17 cells represent a T cell subset that can counteract the function of regulatory T cells (Treg cells) [66]. Nevertheless, alterations in Th17 cell populations within the visceral adipose tissue (VAT) in vivo have not yet been definitively identified. Besides macrophages and B cells, resident dendritic cells (DCs) are also capable of recognizing dying adipocytes. Mechanistic investigations conducted by our research group and others have suggested that the innate functions of DCs may contribute to inflammation induced by obesity. This is due to the activation of DCs by apoptotic debris and free fatty acids through Toll-like receptors (TLRs), a process facilitated by obesity-related factors such as hypoxia and increased lipolysis within the adipose tissue [57,68]. The activation of dendritic cells (DCs) through Toll-like receptors (TLRs) triggers the release of pro-inflammatory cytokines that are associated with adipose tissue inflammation and insulin resistance. While there is substantial evidence supporting the innate role of DCs in inflammation induced by obesity, there is a need for intensified research efforts to comprehend how the adaptive functions of DCs might impact B and T cells during this prolonged inflammatory response.

### 3.3. T Cells

T cells were identified in VAT almost 23 years ago [69]. T cells play a pivotal role in regulating macrophage polarization and are believed to have a regulatory role in modulating inflammation during both insulin-sensitive and insulin-resistant states [6].

Furthermore, the depletion of CD3+ T cells resulted in a reduced inflammation within visceral adipose tissue (VAT), an improved glucose tolerance, and a reduced insulin resistance in diet-induced obese (DIO) mice [70]. T cells are thought to infiltrate visceral adipose tissue (VAT) following B cells, and their role in inflammation related to type 2 diabetes has been extensively investigated in mice [63]. Similar to B cells, T cells are categorized into various subsets based on their phenotype and function. T regulatory cells (Tregs) represent an anti-inflammatory subset of T cells that contribute to maintaining visceral adipose tissue (VAT) homeostasis during the lean state in mice. Tregs have the capacity to mitigate VAT inflammation and insulin resistance. They also secrete IL-10, which helps polarize macrophages toward the anti-inflammatory M2 phenotype, and they can block the production of pro-inflammatory cytokines like IL-6 induced by TNF-α [6,56]. Moreover, in mice, there is a clear and direct relationship between the proportion of T regulatory cells (Tregs) present in visceral adipose tissue (VAT) and the level of insulin sensitivity [71]. Under normal conditions, T regulatory cells (Tregs) make up approximately 30–50% of T cells within visceral adipose tissue (VAT) and exhibit elevated expression of interleukin-10 (IL-10) [71]. On the other hand, the proportion of Treg cells in VAT decrease during the obese state [70–72]. This decline in Tregs within visceral adipose tissue (VAT) is likely attributed to the influx of recruited pro-inflammatory cells. Similar decreases in Tregs have also been observed in humans [73].

The pro-inflammatory T cell subsets that are most closely associated with inflammation in type 2 diabetes are Th1 cells and CD8+ T cells. Generally, diet-induced obese (DIO) mice have approximately three times as many T cells in their visceral adipose tissue (VAT) compared to mice who were fed a standard diet, suggesting an elevated recruitment of T cells in obesity [14]. These T cells are typically located in close proximity to clusters of macrophages and regions of dying (apoptotic/necrotic) adipocytes, which are referred to as crown-like structures (CLSs) [5,53]. The significance of T cells in obesity-induced insulin resistance is underscored by the fact that inhibiting the migration of T cells to visceral adipose tissue (VAT) can prevent insulin resistance in diet-induced obese (DIO) mice [74]. In mice who were fed a high-fat diet, both the percentage and the total number of Th1 cells producing interferon-gamma (IFN-γ) are elevated within the visceral adipose tissue (VAT) [72,75,76]. This scenario has also been found to occur in humans [72,77]. IFN-γ aids in the polarization of macrophages to the M1 phenotype. Th1 cells also secrete IL-12. Th1-deficient IL-12p35 null mice on a high-fat diet have improved insulin sensitivity [72]. Th1 cells are not the only T cell subset implicated in obesity-induced inflammation. CD8+ T cells are found to increase in the VAT of DIO mice after 2 weeks [70]. Most are activated and they colocalize with CLSs [70]. CD8+ T cells help to recruit M1 macrophages as indicated by the reduction in M1 macrophages, IL-1, IL-6, TNF-α, MIP-α, and RANTES in VAT when CD8+ T cells were abolished [70]. Also, CD8+ T cells may function in supporting monocyte differentiation [70]. Although several studies in mice indicate that Th1 cells may be driving inflammation in type 2 diabetes, recent work indicates that Th17 cells may be the most central T cell subset in human type 2 diabetes [78–81]. Indeed, mice lacking the IL-17 gene exhibit increased insulin sensitivity but decreased tolerance to glucose when compared to the control group [82]. Furthermore, the introduction of an anti-IL-17 antibody to insulin-resistant mice induced by angiotensin II resulted in enhanced insulin sensitivity and reduced glucose intolerance [83].

### 3.4. B Cells

B cells have recently been shown to play a role in type 2 diabetes. They consist of two major subsets, B-1 and, more commonly, B-2 cells [6]. B-1 cells are further divided into B-1a cells which produce the majority of natural IgM and B-1b cells, which are involved in the T cell-independent antigen humoral response [84]. B-2 cells on the other hand respond to T cell-dependent antigens. Another subset of B cells, the B-10 cells are IL-10 producing B cells, which are part of a larger subset called B regulatory cells (Bregs) [85]. Much of what is known regarding the role of B cells in type 2 diabetes and visceral fat inflammation is derived from DIO mice studies. B cells are known to be involved in type 2 diabetes because B cell-deficient DIO mice have an increased insulin sensitivity [86]. In the lean state, adipose natural B regulatory cells (Breg and B-10), in addition to spleen-derived B-10 and B-1a cells, are known to secrete IL-10 and protect against insulin resistance [87–89]. However, during obesity, the total number of B cells increases in VAT, as well as the amount of pro-inflammatory B-2 cells [86,90]. Transfer of B-2 cells from DIO mice to DIO B cell null mice deteriorates metabolic status [86]. It has been shown that there is an increased class switching of B cells to IgG and that transfer of IgG from DIO mice, but not from lean mice, worsens insulin resistance [86]. In addition, B cells induce the MHC-dependent secretion of cytokines from T cells such as IFN-γ, which can polarize macrophages to a pro-inflammatory M1 phenotype. Although less about the role of B cells in type 2 diabetes in humans is known, Winer et al. has shown that distinct profiles of autoantibodies are associated with insulin resistance [6,86]. Additionally, B cells from type 2 diabetics secrete less IL-10 in response to TLR2, 4, or 9 stimulations [91]. However, B cells do not induce insulin resistance on their own. The transfer of B cells from DIO mice to DIO mice lacking both T and B cells has little effect on glucose tolerance [86,92]. Therefore, much of B cells’ role in type 2 diabetes may be mediated through T cell dependent mechanisms.

### 3.5. Other Immune Cells

The immune system is very complex and, although much research regarding obesity-induced inflammation has been focused on B cells, T cells, and macrophages, a host of other cellular players have been implicated. Neutrophils have been shown to transiently migrate into VAT and the inhibition of neutrophils improves obesity-induced insulin resistance and macrophage accumulation in mice who were fed a high-fat diet [93,94]. In addition, obesity in response to a high-fat diet increases neutrophil infiltration within a week [94]. These results suggest that, similar to a classic immune response, neutrophils are one of the first cells recruited to the site of “infection” [63]. Eosinophils produce the majority of IL-4 in VAT during the lean state. This IL-4 has been shown to be critical in polarizing macrophages to the M2 phenotype in M2-GFP reporter mice [95]. Conversely, obesity decreases the number of eosinophils in the adipose tissue, indicating that this cell type may be involved in regulating macrophages during obesity-induced inflammation. Mast cells, although strongly associated with allergy and asthma, have been shown to be important in obesity-induced insulin resistance. The deletion of mast cells in a high-fat diet mouse model improves insulin resistance and the reconstitution of mast cells worsens it. However, the reconstitution of mast cells in IL-6 or IFN-γ knockout DIO mice did not worsen insulin resistance. These data indicate that IL-6 or IFN-γ made by mast cells in VAT are important to the development of insulin resistance [96]. NKT cells, which generally recognize lipid antigens presented on CD1d molecules, are found to be increased in VAT in response to a high-fat diet in mice within six weeks [97]. However, CD1d-deficient mice present no differences in insulin resistance and glucose intolerance [98]. Several studies have published contradictory results regarding the role of NKT cells in obesity-induced inflammation. Therefore, more research is necessary to investigate the role of NKT cells and other cellular players in the development of VAT inflammation.

## 4. Systemic Inflammation in Type 2 Diabetes

**Figure 2.**
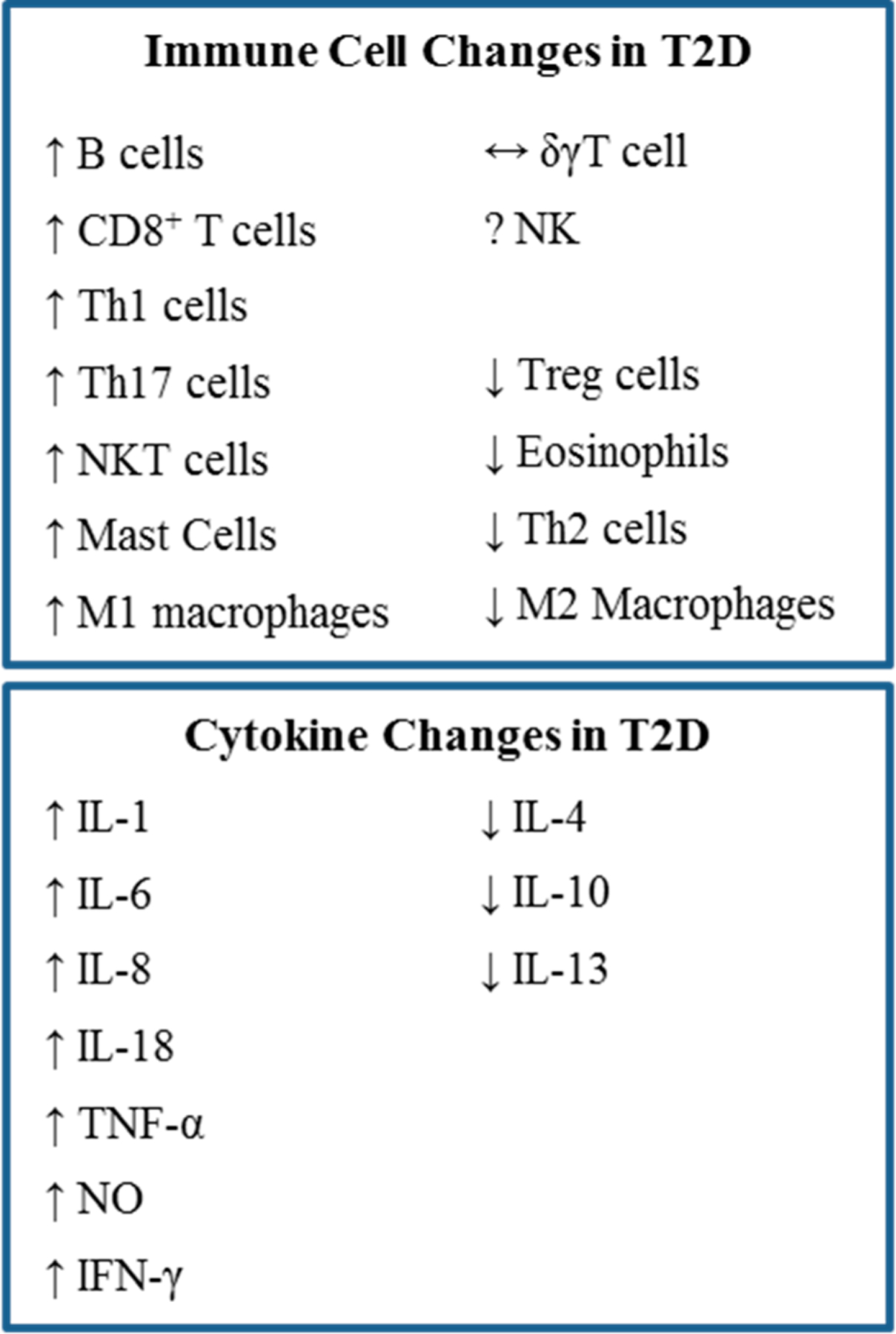
Summary of cellular and cytokine changes during obesity-induced inflammation

Chronic inflammation due to obesity does not only affect the VAT. Increased secretion of pro-inflammatory cytokines by immune cells in the adipose tissue also causes systemic inflammation and insulin resistance in muscle tissue. A summary of these cytokine changes is presented in Figure 2. Although many cytokines and chemokines are involved in obesity-induced inflammation, TNF-α, IFN-γ, IL-6, and especially IL-1β have been closely linked to systemic insulin resistance.

TNF-α was the first pro-inflammatory cytokine to be associated with obesity and insulin resistance [10]. TNF-α is known to inhibit triglyceride deposition and induce lipolysis [99–101]. Similar to non-esterified free fatty acids, TNF-α inhibits insulin-stimulated IRS-1 tyrosine phosphorylation and glucose uptake by GLUT4 [56]. Two studies in patients with rheumatoid arthritis undergoing infliximab (monoclonal antibody against TNF-α) therapy showed a rapid beneficial effect on insulin resistance and sensitivity [102,103]. However, several follow up studies and other studies using neutralizing anti-TNF-α anti-bodies such as Etanercept did not show any improvement in insulin sensitivity [104,105]. Although conflicting results have been found using TNF-α as a target for the treatment of type 2 diabetes and obesity-induced inflammation, this cytokine is important because it introduces a link between inflammation, obesity, and insulin resistance.

IFN-γ is another pro-inflammatory cytokine shown to be related to insulin resistance. Glucose intolerance and insulin sensitivity are increased in IFN-γ-deficient DIO mice [76]. In addition, IFN-γ reduces the insulin-induced uptake of glucose in adipocytes and can polarize macrophages to the M1 phenotype [106–108]. Lastly, IFN-γ can induce the expression of T cell and monocyte chemoattractant proteins such a RANTES and MCP-1 and 2 from an adipocyte cell line [76]. IL-6 is a classical pro-inflammatory cytokine that has also been implicated in metabolic inflammation. In obesity, IL-6 is increased [109]. This increased IL-6 is associated with increases in systemic Th17 cells in DIO mice [110]. Although the role of IL-6 in insulin resistance is controversial, it has been targeted as a potential treatment for metabolic inflammation. For example, rheumatoid arthritis patients with diabetes experienced an HbA1c drop when treated with the anti-IL-6 antibody tocilizumab [111].

IL-1β has recently become the most prominent cytokine attributed to inducing insulin resistance. Once IL-1β is produced, it must be processed into its mature form by caspase 1, which is activated by the NOD-, LRR-, and pyrin domain-containing 3 (NLRP3). IL-1β expression is upregulated during obesity and is strongly related to a high-fat diet (Table 1). Our laboratory has shown that it is positively correlated to the amount of saturated FA intake, specifically dietary PA (Table 1). Additionally, we have shown that PA can directly induce the secretion of IL-1β from dendritic cells by binding TLR4 [68]. Free FAs may also directly activate the NLRP3 inflammasome [112]. High concentrations of glucose promote the secretion of IL-1β from β-cells by the dissociation of thioredoxin-interacting protein (TXNIP) from its inhibitor thioredoxin (TXR) [113]. This results in the activation of NLRP3. IL-1β induces the production of a wide range of cytokines and chemokines implicated in insulin resistance and type 2 diabetes such as CC-chemokine ligand 2 (CCL2), CCL3, and CXC-chemokine ligand 8 (CXCL8) [113]. These chemokines lead to the recruitment of macrophages. Because of IL-1β’s role in insulin resistance and obesity-induced inflammation, it has become the target of several clinical trials. Therapeutics against IL-1β show promise for treating type 2 diabetes. Patients with type 2 diabetes who were treated with Anakinra, Interleukin-1 receptor antagonist (IL-1Ra), for 13 weeks had reduced HbA1c, reduced systemic IL-6, IL-17, and C-reactive peptide, and an increased secretion of C-peptide [114]. These effects lasted up to 39 weeks post withdrawal from the medication [115]. However, insulin sensitivity did not change. In summary, blocking IL-1 improved β-cells function and reduced markers of systemic inflammation.

## 5. Novel Concepts in Diet-Induced Inflammation and Type 2 Diabetes

It is now widely accepted that type 2 diabetes is more than just a metabolic disease. Insulin resistance seems to be driven by high-fat diets and obesity in an inflammation-dependent manner. The involvement of adipocyte MHCII and Fc receptors (FcRs), autoantibody signatures, and restricted T cell repertoires in obese VAT suggests that inflammation associated with insulin resistance is not non-specific [118]. Clearly, the net pro-inflammatory environment in obese mice VAT (IL-1β, IL-12, IL-18, and TNF-α) favors a Th1 response. Th1, CD8+ T cells, and likely Th17 cells in humans induce and perpetuate macrophage activation during obesity-induced inflammation and B cells support this inflammation by secreting antibodies which can activate macrophage FC receptors [6,86,119,120]. The emerging role of specific responses from the adaptive immune system suggests that antigen-presenting cells may play a key role in the development of obesity-induced inflammation.

Dendritic cells, professional antigen-presenting cells, which can activate naïve T cells, have been used to demonstrate how constituents of a high-fat diet, namely palmitic acid, could directly contribute to insulin resistance and obesity-induced inflammation. Palmitic acid elicits a robust IL-1β response from dendritic cells in a TLR4 dependent manner [68]. IL-1β can induce insulin resistance by affecting insulin receptor phosphorylation and insulin receptor substrate 1 expression [121]. In addition, there is now evidence that FAs could directly stimulate the inflammasome, also important in the development of insulin resistance and type 2 diabetes [112,122,123].

Up until recently, the mechanism by which saturated FAs activate TLR4 has been elusive. Our laboratory has recently demonstrated that PA is an actual TLR4 ligand [68]. Further research will be necessary to determine which other saturated FAs can bind TLR4, in addition to understanding how polyunsaturated fatty acids block TLR4 signaling [45,112]. Lastly, the emerging role of B cells and antibodies in obesity-induced inflammation leads to the concept that diet and lipids may affect B cell function in type 2 diabetes [86]. Our laboratory has recently identified IgG antibodies in human serum that can recognize non-esterified saturated FAs [68]. These antibodies seem to be elevated and more frequent in populations with unmanaged diabetes. Although the role of these antibodies is unknown, their presence indicates lipids may somehow be involved in the immune responses seen in type 2 diabetes.

## 6. Summary and Conclusions

The longstanding understanding that a high-fat diet plays a significant role in the development of type 2 diabetes has been supplemented by recent insights into the direct impact of diet and obesity on the inflammation associated with this condition. Nevertheless, the intricate interactions between dietary components and our immune system remain poorly elucidated. Non-esterified long-chain saturated fatty acids, such as palmitic acid (PA), have established associations with obesity, insulin resistance, and inflammation. It is now evident that saturated fatty acids possess the potential to directly influence immune cells, initiating a series of events that ultimately contribute to insulin resistance. Consequently, it is crucial to gain a deeper understanding of the mechanisms through which free fatty acids interact with the immune system, with the aim of developing effective treatments for inflammation induced by obesity and type 2 diabetes.

## Figures and Tables

**Table 1.** Serum IL-1β from Hispanic patients with type 2 diabetes (N = 7) correlates with variables related to dietary fat intake. IL-1β was measured in serum samples obtained from participants in the En Balance diabetes intervention program and correlated with dietary intake via the University of Arizona Southwest Food Frequency Questionnaire [116,117].

Variables	Spearman’s Correlation	*p*-Value
Total dietary fat intake (g)	0.821	0.023
Energy (kcal)	0.929	0.003
Dietary Cholesterol (mg)	0.786	0.036
Saturated fatty acids (g)	0.857	0.014
10:0 Capric acid (g)	0.786	0.036
16:0 Palmitic acid (g)	0.893	0.007
18:0 Stearic acid (g)	0.821	0.023
Monounsat. fatty acids (g)	0.821	0.023
18:1 Oleic Acid (g)	0.821	0.023

## Data Availability

Not Applicable.
